# Cover crop species influences soil fungal species richness and community structure

**DOI:** 10.1371/journal.pone.0308668

**Published:** 2024-09-12

**Authors:** Ephantus J. Muturi, Christopher A. Dunlap, William L. Perry, Robert L. Rhykerd

**Affiliations:** 1 U.S. Department of Agriculture, Agricultural Research Service, National Center for Agricultural Utilization Research, Crop Bioprotection Research Unit, Peoria, Illinois, United States of America; 2 School of Biological Sciences, Illinois State University, Normal, Illinois, United States of America; 3 Department of Agriculture, Illinois State University, Normal, Illinois, United States of America; Universidade Federal de Minas Gerais, BRAZIL

## Abstract

Despite the well documented link between cover cropping and soil microbiology, the influence of specific cover crop species on soil microbes remains poorly understood. We evaluated how soil fungal communities in a no till system respond to four cover crop treatments: no cover crop (REF), cereal ryegrass (CRYE), wild pennycress (WPEN), and a mix of pea, clover, radish, and oat (PCRO). Soil samples were collected from experimental plots following termination of cover crops from depths of 0–2 cm and 2–4 cm where cover crops had significantly increased soil organic matter. There was no significant interaction between soil depth and cover crop treatment on either alpha diversity or beta diversity. All cover crop treatments (CRYE, PCRO, and WPEN) enhanced soil fungal richness but only CRYE enhanced soil fungal diversity and altered the fungal community structure. Soil depth altered the fungal community structure but had no effect on fungal diversity and richness. Genus *Fusarium* which includes some of the most economically destructive pathogens was more abundant in REF and PCRO treatments compared to CRYE and WPEN. In contrast, genus *Mortierella* which is known to promote plant health was more abundant in all cover crop treatments relative to the REF. These findings demonstrate that cover cropping can increase soil fungal species richness and alter fungal community structure, potentially promoting the abundance of beneficial fungi and reducing the abundance of some plant pathogens within the genus *Fusarium*. These effects are dependent on cover crop species, a factor that should be considered when selecting appropriate cover crops for a particular cropping system.

## Introduction

The world’s population is increasing at an unprecedented rate and is projected to reach 9.7 billion by 2050. Substantial increase in food production is needed to keep up with the rapidly growing population at a time when the world’s arable land is shrinking [1]. Conventional agricultural practices such as genetic selection of high yielding crops, irrigation, and use of chemical pesticides and fertilizers, have kept pace with the rising food demand, but have also been associated with environmental challenges such as soil degradation, soil erosion, water pollution, and increased emission of greenhouse gases [2]. These challenges have stimulated interest in adopting agroecological practices that promote efficient use of resources to increase agricultural production while building healthy, resilient, and sustainable agroecosystems.

Cover cropping provide soil cover between growing seasons and has gained traction as a key component of sustainable agriculture. Cover crops can be annual, biennial, or perennial legume or non-legume plants grown as single species or a mixture of species. These crops promote agricultural sustainability by reducing nutrient leaching and soil erosion, suppressing weeds and soil-borne pathogens, enhancing populations of beneficial insects, and increasing carbon sequestration in the soil [3–12]. Cover crops also promote soil health by enriching the soil with organic matter and nutrients, enhancing soil porosity and water-holding capacity, and increasing microbial diversity, biomass, and activity [11, 13]. Each cover crop provides a subset of benefits based on its functional traits. For example, legumes boost moisture retention and increase nitrogen availability in the soil through symbiotic association with microorganisms, brassicas suppress soilborne pathogens, increase crop yield, and improve soil properties, while cereal cover crops improve soil fertility and capacity for water retention [14–17].

In the Midwestern U.S., corn and soybean farming is a major non-point source of nitrate pollution in waterways, and farmers are increasingly encouraged to adopt conservation practices such as cover cropping to promote soil and water sustainability [18]. Cereal rye (*Secale cereale* L.), which has an extensive root system that promotes nitrogen retention by penetrating deep soil layers [19], is the most common cover crop grown by corn and soybean farmers in this region. This is due to cereal ryes affordability, ease of establishment in the fall after corn and soybean harvest, potential to accumulate high biomass, and ability to survive the winter and to suppress winter weeds [20]. Other plants promoted as cover crops include annual ryegrass, hybrid rye, pennycress, hairy vetch, pea, clover, radish, oats, their mixtures, among others [18]. However, research comparing the influence of specific cover crop species on soil health is limited. This knowledge is critical for the selection of appropriate cover crops for a particular cropping system as some cover crops may be more suited for some soil and agroecological environments than others.

Fungi consist of morphologically, functionally, and phylogenetically diverse group of organisms with many distinct trophic guilds. They attract significant interest as major pathogens of crops but also promote soil and plant health by suppressing pests and pathogens, promoting nutrient cycling and decomposition of organic matter, improving soil structure, and enhancing plant resilience to abiotic stresses such as drought, salinity, soil acidity, high temperatures, and nutrient deficiency [21–23]. Some fungal groups also promote plant growth by producing growth-promoting substances or establishing symbiotic associations that aid the host plant to acquire nutrients from the soil [24, 25]. Cover crops have been shown to increase soil microbial diversity, biomass, and activity, and to support large populations of beneficial microbes [13, 26–28]. These effects may differ across cover crop species since different plants produce distinct bioactive substances that may promote or suppress growth of specific microbial groups [14–17, 29]. For example, glucosinolates produced by plants in the family Brassicaceae are hydrolyzed by enzyme myrosinase into isothiocyanates which have antifungal properties [30]. There is also evidence that root-associated fungi show preference for certain plant species [31, 32]. However, our understanding of how soil fungal communities respond to different cover crop species is limited. In addition, although it is well documented that soil fungal community structure differs across soil depths, and that soil fungal diversity and richness decreases with increasing soil depth, our understanding of how soil depth affects fungal communities under different cover crop species is limited [33–35]. Cover crops differ in their rooting systems which may influence how they interact with soil microbes at different soil depths. For example, cereal cover crops have extensive rooting systems that penetrate deeper in the soil compared to legume cover crops. Addressing these knowledge gaps is essential for the development of effective strategies to enhance the physical, chemical, and biological properties of managed soils.

In this study, we used high-throughput sequencing of the ITS1 region of the ribosomal RNA gene to explore the short-term effect of 4 cover crop treatments (no cover crop (REF), cereal rye (CRYE), wild pennycress (WPEN) and a mix of pea, clover, radish, and oat (PCRO) on diversity and community structure of fungal communities in agricultural soils. We tested the hypotheses that 1) cover cropping will increase soil fungal diversity and alter fungal community structure, 2) fungal community structure and diversity will differ between the two soil depths with the upper soil depth (0–2 cm) having higher microbial diversity compared to lower soil depth (2–4 cm), and 3) the effect of cover crop on soil fungal communities will vary between the two soil depths.

## Materials and methods

### Experimental design and soil sampling

The study was conducted on a Catlin, Flanagan, and Drummer soil series at the Illinois State University research farm at Lexington, IL. (40.674641, -88.783492). This study complied with all relevant regulations and no permits were required. Historically, the plots have been in a corn-soybean rotation and winter fallow, typical of the U.S. Midwest. The experimental design was a randomized complete block design with four blocks. At the beginning of the experiment on October 17, 2020, subplots with dimensions of 4.6 m x 12.2 m within each block were randomly assigned and seeded with one of the four cover crop treatments. These included a winter fallow reference plot (REF), cereal ryegrass (CRYE), wild pennycress (WPEN), and a mixture of pea, clover, radish, and oat (PCRO). The cover crops were selected for the following reasons. Cereal rye is the most commonly used cover crop in the Midwest and is easily established. Pennycress was selected because it is being developed into a winter oil-seed cash crop [36]. The PCRO mix was selected because it is composed of a diverse group of winter cover crops. The oats and radish typically germinate in the fall and provide ecosystem services until they are terminated by a frost. The peas and clover typically germinate in the spring and because they are legumes, can fix atmospheric N, improving soil fertility. The cover crops were planted using a drill at rates of 56.0 kg ha^-1^ for CRYE, 5.6 kg ha^-1^ for PCRO, and 16.8 kg ha^-1^ for WPEN. The summer cash crops were soybean in 2021 and corn in 2022. No fertilizers were applied to the cover crop plots or soybeans. Glyphosate was used to terminate the cover crops and applied to the reference plot at the same time for weed control. Corn was fertilized with nitrogen before tasseling during the summer of 2022 at a rate of 90 kg ha^-1^. Soil samples were collected using a handheld soil probe on June 13, 2023, at two soil depths (0–2 cm and 2–4 cm), placed on dry ice, and transported to the USDA Laboratory in Peoria, IL where they were preserved at -80 °C until further processing.

### Soil DNA extraction and sequencing

Soil samples were freeze dried and 20 g of each sample was homogenized in a blender (Waring Inc) for 5–15 s until finely ground. DNA was extracted from 200 mg of this soil mixture using DNeasy PowerLyzer PowerSoil Kit (Qiagen Inc) following the manufacturer’s protocol. DNA concentration and quality were determined with a NanoDrop (Thermo Fisher Scientific). Polymerase chain reaction (PCR) amplification of the ITS1 region of the ribosomal RNA gene was performed using primer pair ITS1F and ITS2 [37] that were added at the end of Illumina overhang adapter sequences. The 30 μL PCR reaction consisted of 15 μL of Amplitaq Gold 360 master mix (ThermoFischer Scientific Inc), 1.5 μL of each primer, and 12 μL of template DNA. Amplification conditions were an initial denaturation at 95 ◦C for 10 min, 35 cycles of 95 ◦C for 30 s, annealing at 58 ◦C for 30 s and extension at 72 ◦C for 60s. PCR amplicons were checked on a 2% agarose gel using gel electrophoresis and purified using Ampure XP beads (Beckman Coulter, USA). Index PCR to attach dual indices and Illumina sequencing adapters was conducted as described before [38]. DNA libraries were cleaned and normalized using a SequalPrep^™^ normalization plate (Thermofisher inc, Waltham, MA). The samples were pooled, and the library quantified with a Kapa library quantification kit (Kapa Biosystems Willington, MA). Paired-end sequencing (2 x 300 bp) of the libraries was performed in-house on an Illumina MiSeq system using MiSeq reagent kit V3.

### Measurement of soil chemical and physical properties

Selected physical and chemical properties of soil from the experimental plots were quantified and the resulting data used to relate soil properties to fungal community data. Soil samples were shipped to the United Soils Inc. (USI), laboratory in Fairbury, IL for testing analysis. USI’s quality control and assurance program includes analysis of known soil standards, chemical standards, and customer duplicates. Further, they participate in three separate industry proficiency and certification programs to ensure their test quality adheres to industry standards and have been recognized for meeting these program standards. USI analyzes soil samples following procedures described in the Recommended Chemical Soil Test Procedures for the North Central Region [39]. Soil samples were analyzed for pH, buffer pH (bpH), soil organic matter (OM), cation exchange capacity (CEC), soil extractable NO_3_-N (NO_3_), soil extractable NH_4_-N (NH_4_), soil total N (STN), soil total C (STC), P, K, Ca, Mg, Na, S, Zn, Fe, Mn, Cu, and B. Soil samples for these analyses were collected separately from the samples used for fungal community analysis and transported to the Department of Agriculture at Illinois State University’s research laboratory where they were dried in an oven at 40°C, ground with a mechanical grinder, and passed through a 2-mm sieve. The sieved soil samples were then transported to the USI laboratory for chemical and physical analysis. A pH meter and glass electrode were used to measure soil pH in both water and Sikora buffers. Soil pH was measured from a slurry of 10 g soil and 10 ml of either distilled water or Sikora buffer. Soil total carbon and nitrogen were measured using a C:N analyzer from approximately 1 g of soil. Soil extractable NO_3_-N (NO_3_) was measured calorimetrically using a spectrophotometer and NH_4_-N (NH_4_) was measured using an ammonia analyzer from 2 g of soil. P, K, Ca, Mg, S, Fe, Zn, Cu, B, Mn, and Na were measured using inductively coupled plasma (ICP) mass spectrometry from 2 g of soil. CEC was measured by summation of the measured cations.

### Data analysis

Demultiplexed raw sequences were imported into QIIME2 (version 2023.2) and inspected for quality. The divisive amplicon denoising algorithm (DADA2) plugin in QIIME2 was used to filter and trim the reads, dereplicate the filtered reads, learn the error rates, remove chimeric sequences, merge forward and reverse reads, and create the sequence table [40]. Denoise-paired command was implemented with default options except for the following parameters: trim-left-f 22, trim-left-r 20, trunc-len-f 220, trunc-len-r 200. Taxonomy assignment for each amplicon sequence variant was accomplished using feature classifier classify-sklearn with UNITE database v7.2 [41].

Statistical analysis was conducted using R 4.1.0 statistical package. Data was imported into R statistical package and converted into a phyloseq class object using phyloseq R package [42]. ASVs not seen more than 5 times in at least 5% of the samples were removed before downstream analysis to reduce the problem of spurious OTUs [43]. Three samples with very low read counts (less than 10,140 reads) were further excluded from the analysis. These included samples B3CRYE_0_2, B3CONT_0_2, and B2PCRO_2_4 with 66, 1035, and 5927 reads, respectively). The data was transformed to relative abundance and non-metric multidimensional scaling analyses of Bray-Curtis and weighted UniFrac distance matrices were performed using metaMDS function in vegan R package (hereafter vegan) to determine similarity in fungal communities among cover crop treatments and soil depths [44]. Permutational multivariate analysis of variance (PERMANOVA) using the “adonis2” function in vegan was used to test the effects of cover crop treatment and soil depth on fungal communities [44]. Pairwise comparisons to identify treatments that were significantly different was achieved using pairwiseAdonis R package [45]. DESeq2 R package was used to identify ASVs that differed significantly between soil depths (0–2 cm vs 2–4 cm) and among cover crop treatment pairs that were significantly different [46].

For alpha diversity analysis, samples were rarefied to an even depth of 10,140 sequences before observed ASVs (species richness), Shannon diversity, and Faith’s phylogenetic diversity (PD) indices were calculated for each sample using “vegan” package. Data were checked for normality using Shapiro-Wilk normality test and Shannon diversity index data were log-transformed before analysis because unlike observed ASVs and Faith’s PD data, it did not meet the assumptions of normal distribution. Data were analyzed using two-way ANOVA and differences in means between treatments evaluated using Tukey’s HSD test.

MANOVA was used to evaluate the effect of cover crop treatment and soil depth on soil physical and chemical properties. Data were log(x + 1) transformed to meet the assumptions of normality. Follow up univariate ANOVAs and TukeyHSD tests were used to evaluate the means that differed significantly between treatments. Redundancy analysis (RDA) with Mote Carlo permutation test (999 permutations) was used to link the changes in soil fungal community with soil physical and chemical properties. Vector length of RDA biplot was used to reflect the relative importance of soil physical and chemical properties in discriminating the fungal communities. Spearman’s rank correlation was used to evaluate the correlations between the relative abundances of different fungal genera and soil variables.

## Results

**Fig 1:**
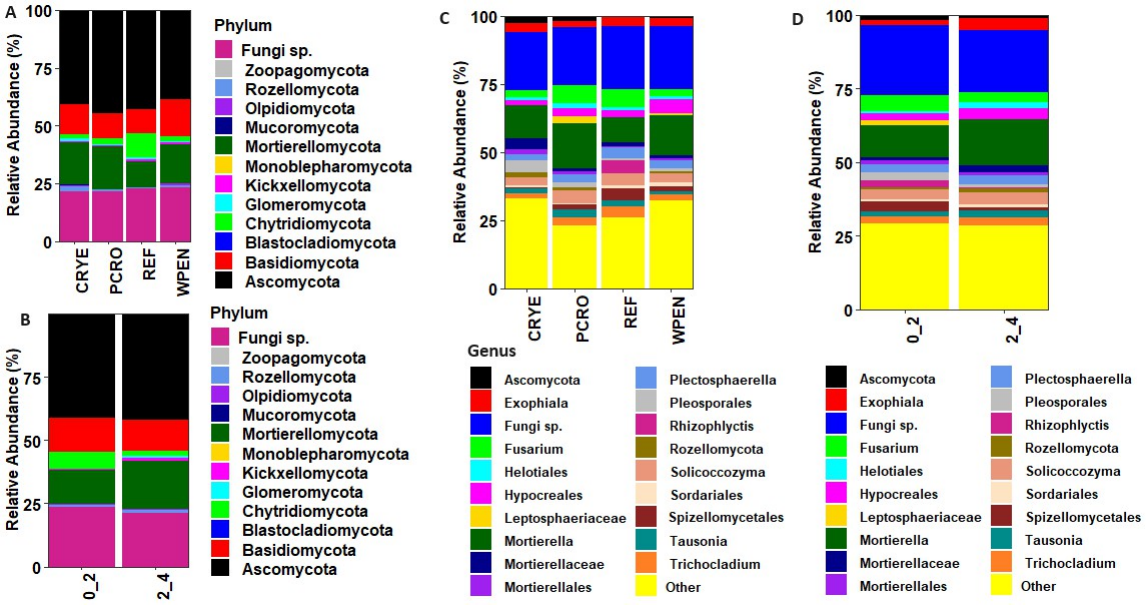
Relative abundance of soil fungal communities at phylum and genus levels in relation to cover crop treatments and soil depth.

A total of 737,314 sequences were obtained. After quality filtering, three samples with less than 10,140 reads were excluded from further analysis. The number of sequences in the remaining samples ranged from 10,140 to 46108 with a median of 24,260. A total of 994 amplicon sequence variants (ASVs) were identified across all samples. The six most abundant phyla across all cover crop and soil depth treatments were Ascomycota (41.7%), unclassified fungi (22.3%), Mortierellomycota (16.1%), Basidiomycota (12.7%), Chytridiomycota (4.2%), and Rozellomycota (1.0%). The remaining phyla including Kickxellomycota, Glomeromycota, Olpidiomycota, Mucoromycota, Monoblepharomycota, Zoopagomycota, and Blastocladiomycota accounted for 1.9% of the total reads. The relative abundance of some phyla varied markedly between cover crop species (Fig 1A). The relative abundance of Ascomycota and was lowest in WPEN (38.4%), intermediate in CRYE (40.6%) and REF (43.0%) and highest in PCRO (44.7%). The relative abundance of Mortierellomycota was lowest in REF and highest in CRYE and PCRO. Basidiomycota relative abundance was lowest in REF (10.5%) and PCRO (10.9%), intermediate in CRYE (13.4%), and highest in WPEN (16.2%). Chytridiomycota was more abundant in the REF relative to the other cover crop treatments. Soil depth also influenced the relative abundance of fungal phyla (Fig 1B). The relative abundances of Ascomycota and Basidiomycota were similar between the two soil depths. Conversely, Mortierellomycota was more abundant at 2–4 cm depth compared to 0–2 cm depth while Chytridiomycota was more abundant at 0–2 cm depth compared to 2–4 cm depth.

At the genus level, the top 10 taxa across cover crop treatments included unclassified fungi (22.3%), *Mortierella* (13.2%), *Fusarium* (4.5%), *Solicoccozyma* (3.8%), *Exophiala* (3.0%), *Plectosphaerella* (3.0%), *Hypocreales* (2.9%), *Trichocladium* (2.7%), *Spizellomycetales* (2.2%), and *Tausonia* (2.0%). The relative abundance of some taxa varied markedly between cover crop treatments (Fig 1C). Order Mortierellales consisting of unclassified Mortierellales, unclassified Mortierellaceae and genus *Mortierella* was more abundant in cover crop treatments compared to the REF. *Fusarium* was more abundant in REF and PCRO treatments compared to CRYE and WPEN treatments. Order Hypocreales was more abundant in WPEN compared to the other treatments while order Pleosporales was more abundant in CRYE relative to the other treatments. Order Spizellomycetales abundance was highest in REF and lowest in CRYE. With regard to soil depth, order *Hypocreales*, *Plectosphaerella*, *Tausonia*, *Solicoccozyma*, order Sordariales, and order Mortierellales was more abundant at 2–4 cm soil depth while *Fusarium*, family Leptosphaeriaceae, orders Pleosporales and Spizellomycetales were more abundant at 0–2 cm soil depth (Fig 1D).

**Fig 2:**
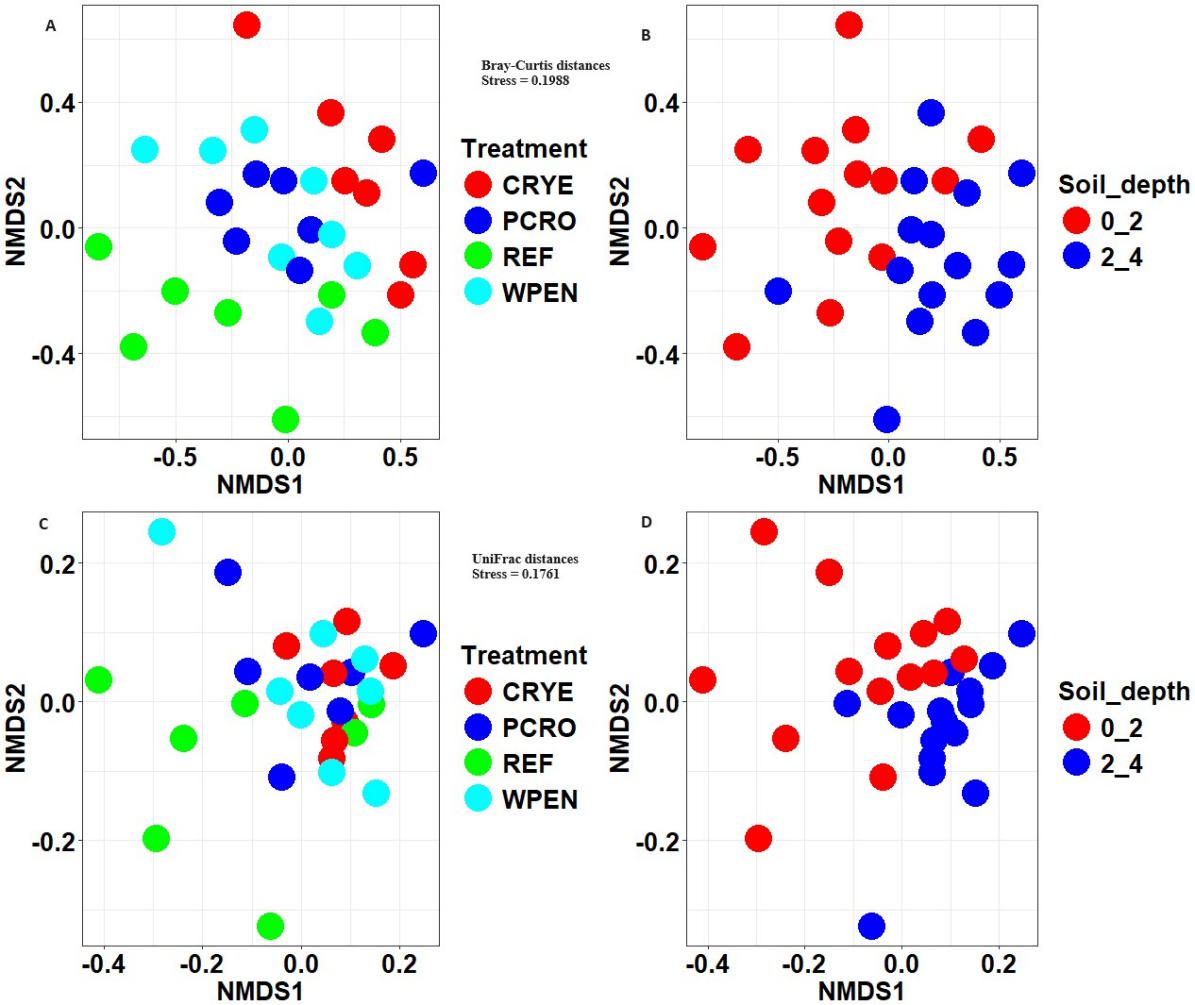
Non-metric multidimensional scaling (NMDS) of Bray-Curtis (A, B) and UniFrac (C, D) distances between fungal communities from 4 cover crop treatments (A and C) and two soil depths (B and D).

ASV richness (observed ASVs), Shannon diversity index, and Faith’s PD were significantly influenced by cover crop treatment but not soil depth or their interaction (Tables 1 and 2). All cover crop treatments had significantly higher ASV richness compared to the REF treatment. Shannon diversity index and Faith’s PD followed a similar trend but only CRYE cover crop treatment had a significantly higher Shannon diversity index and Faith’s PD compared to REF. Non-metric multidimensional scaling with Bray Curtis and UniFrac distance matrices revealed some separation between cover crop treatments and soil depth (Fig 2). PERMANOVA analysis for both distance matrices revealed significant effect of cover crop treatment and soil depth but not their interaction on fungal communities (Bray Curtis distance, cover crop treatment: F = 1.7911, *P* = 0.001; soil depth: F = 2.5415, *P* = 0.001, interaction: F = 0.5841, *P* = 1.000, UniFrac distance, cover crop treatment: F = 1.8544, *P* = 0.003; soil depth: F = 3.7747, *P* = 0.001, interaction: F = 1.452, *P* = 0.452). Pairwise contrasts for Bray Curtis distance matrix revealed significant differences between REF vs CRYE, CRYE vs PCRO, and CRYE vs WPEN cover crop treatments. Similar comparisons for UniFrac distance matrix revealed that only REF vs CRYE comparisons differed significantly after Bonferroni correction for multiple comparisons.

**Table 1:** Soil fungal diversity and richness (mean ± SE) in relation to cover crop treatment.

Treatment	Observed ASVs	Shannon diversity index	Faith’s PD
REF	218.71 ± 4.61^a^	4.14 ± 0.04^a^	55.42 ± 1.24^a^
CRYE	319.29 ± 4.06^b^	4.63 ± 0.02^b^	72.19 ± 0.57^b^
PCRO	283.43 ± 7.87^b^	4.35 ± 0.05^ab^	67.20 ± 1.18^ab^
WPEN	286.00 ± 5.34^b^	4.37 ± 0.03^ab^	67.38 ± 1.44^ab^

**Table 2:** Two-way ANOVA test for the effect of cover crop and soil depth on soil fungal richness (observed ASVs), Shannon diversity and Faith’s PD index.

Variable	Treatment	Df	F	*P* values
**Observed ASVs**	Treatment (T)	3	7.445	**0.0014**
	Soil depth (D)	1	0.006	0.9371
	T x D	3	1.414	0.2666
	Residual	21		
**Shannon**	Treatment (T)	3	4.671	**0.0119**
	Soil depth (D)	1	1.426	0.2458
	T x D	3	1.484	0.2477
	Residual	21		
**Faith’s PD**	Treatment (T)	3	4.982	**0.00914**
	Soil depth (D)	1	1.023	0.32322
	T x D	3	1.388	0.27405
	Residual	21		

**Fig 3:**
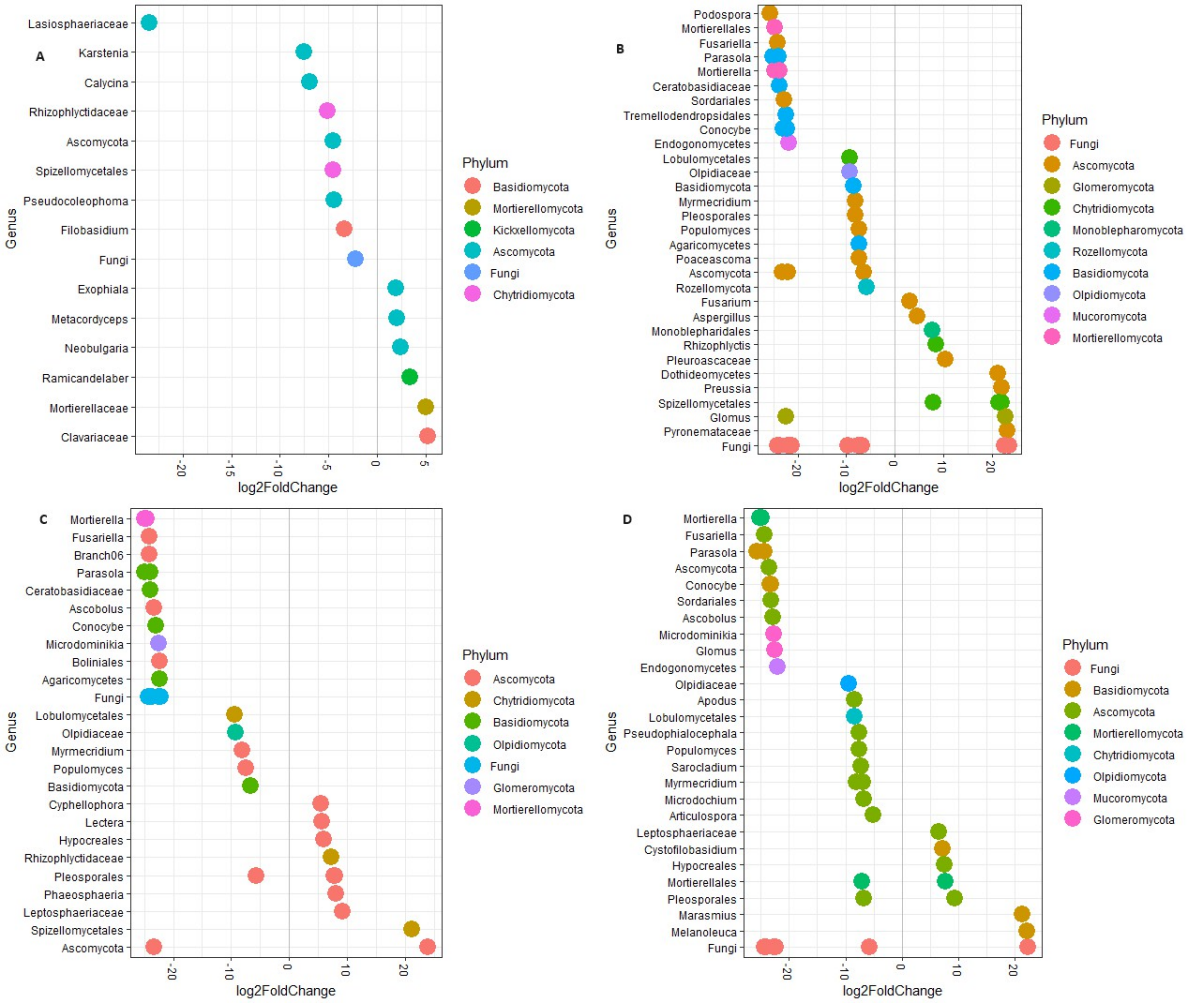
Plots of differentially abundant fungal taxa between A) 2–4 cm vs 0–2 cm soil depth, B) REF vs CRYE, C) CRYE vs PCRO, and D) CRYE vs WPEN.

Differential abundance analysis using DESeq2 package in R was used to investigate the fungal genera that were differentially abundant between the two soil depths and between cover crop treatments that had significantly different fungal community structure (REF vs CRYE, CRYE vs PCRO, and CRYE vs WPEN). Fifteen fungal taxa from Ascomycota, Basidiomycota, Chytridiomycota, Mortierellomycota, Kickxellomycota, and Unclassified fungi, were differentially abundant between the 0–2 cm and 2–4 cm soil depths (Fig 3A). Six of the 15 taxa (e.g. Clavariaceae, Mortierellaceae and *Metacordyceps*) were significantly abundant in 2–4 cm soil depth relative to 0–2 cm soil depth while 9 taxa (e.g. Lasiosphaeriaceae, *Karstenia*, and Spizellomytales) were significantly abundant in 0–2 cm depth relative to 2–4 cm depth (Fig 3A). For the cover crop treatments, 48, 34 and 38 taxa were differentially abundant between CRYE vs REF, CRYE vs PCRO and CRYE vs WPEN, respectively (Fig 3B–3D). Fourteen taxa including *Fusarium*, *Aspergillus*, and Spizellomycetales were significantly abundant in REF relative to CRYE while 34 taxa including *Mortierella* and *Sordariales* were significantly abundant in CRYE relative to REF. Of the 34 taxa that were differentially abundant between CRYE and PCRO, 10 including *Spizellomycetales* and Leptosphaeriaceae were significant abundant in PCRO relative to CRYE while 24 taxa including *Mortierella* and *Conocybe* were significantly abundant in CRYE relative to PCRO. For the 38 taxa that were differentially abundant between CRYE vs WPEN, 8 including *Marasmius* and *Melanoleuca* were significantly abundant in WPEN relative to CRYE and 30 including *Mortierella*, Sordariales and *Glomus* were significantly abundant in CRYE relative to WPEN.

**Fig 4:**
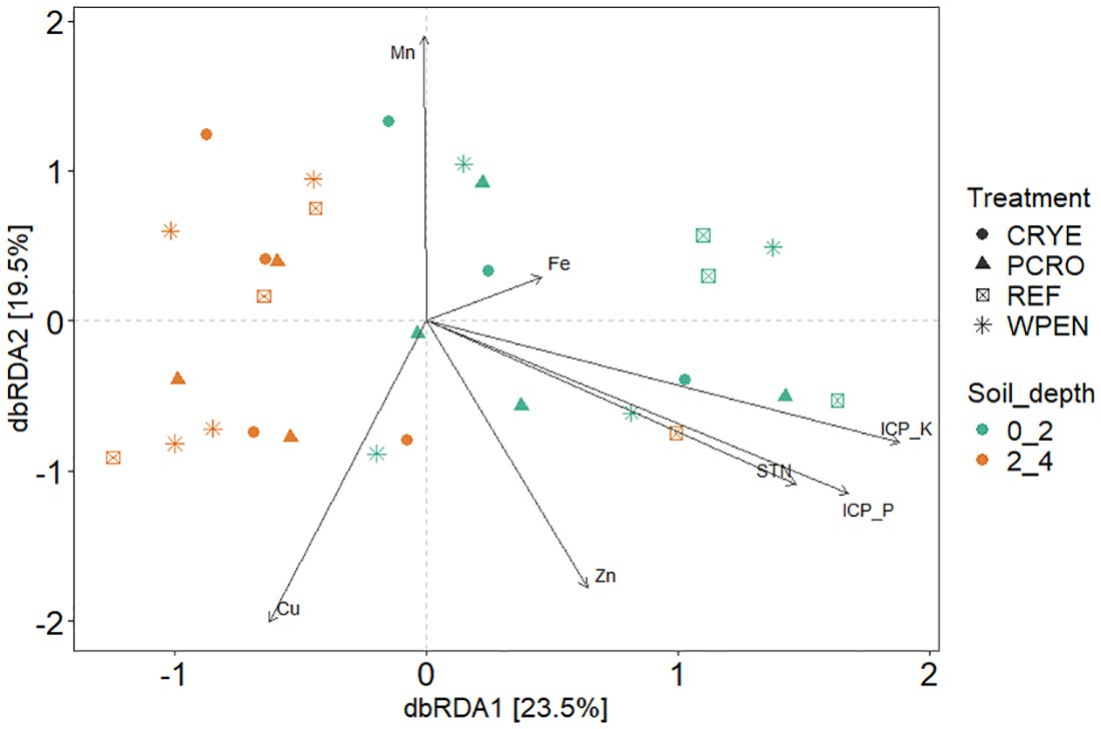
Redundancy analysis (RDA) based on ASV data and soil variables. Arrows indicate the direction and magnitude of soil variables associated with fungal communities. The length of an arrow-line indicates the strength of the relationship between microbial community and soil variable.

MANOVA analysis revealed that soil properties were significantly influenced by soil depth (Pillai = 0.989, df = 4, 18, F = 20.8031, *P* = 0.0048) but not cover crop treatment (Pillai = 2.475, df = 18, 54, F = 1.5697 *P* = 0.1463) or interaction between soil depth and cover crop treatment (Pillai = 2.0187, df = 18, 54, F = 0.6857, *P* = 0.8568). Four of the 18 soil parameters were significantly influenced by soil depth (Table 3). ICP-P, ICP-K, STN, and STC were significantly higher at 0–2 cm depth compared to 2–4 cm. RDA revealed 7 soil variables that had significant correlations with soil fungal community changes. These included Fe, Cu, Zn, Mn, ICP_P, ICP_K, and STN (Fig 4). None of the fungal ASVs under cover crop treatments were significantly associated with soil variables after correction for multiple comparisons. In contrast, 4 significant associations between fungal ASVs and soil variables were detected at the two soil depths. Unclassified Helotiales was negatively associated with ICP_K (r = -0.564, *AdjP* = 0.046) while *Leptospora* (r = 0.547, *PAdjP* = 0.046) and *Neosetophoma* (r = 0.545, AdjP = 0.046) were positively associated with ICP_K. Another *Neosetophoma* sp. was positively associated with ICP_P (r = 0.556, *AdjP* = 0.046).

**Table 3:** Soil characteristics (mean ± SE) and 0–2 cm and 2–4 cm soil depth.

Variable	0–2 cm depth	2–4 cm depth	Df	F value	P
Fe	132.41 ± 3.90	129.85 ± 2.86	27	0.2857	0.5973
Zn	6.56 ± 0.56	6.66 ± 0.50	27	0.0163	0.8992
Cu	1.70 ± 0.11	2.03 ± 0.13	27	3.5914	0.0688
B	0.66 ± 0.02	0.67 ± 0.02	27	0.1058	0.7475
S	9.98 ± 0.66	9.92 ± 0.69	27	0.0037	0.9517
Mn	73.65 ± 7.32	64.48 ± 6.63	27	0.8664	0.3602
ICP_P	71.10 ± 5.24	52.14 ± 5.69	27	5.9558	**0.0215**
ICP_K	337.74 ± 18.66	247.34 ± 11.08	27	17.9160	**0.0002**
ICP_Mg	315.89 ± 18.97	331.27 ± 18.37	27	0.3392	0.5651
ICP_Ca	2535.21 ± 162.13	2973.13 ± 163.95	27	3.5954	0.0687
ICP_Na	16.99 ± 0.94	16.86 ± 0.67	27	0.0122	0.9129
pH	6.39 ± 0.03	6.46 ± 0.05	27	1.2153	0.2800
bpH	6.90 ± 0.03	6.96 ± 0.02	27	3.5911	0.0688
CEC	18.47 ± 1.09	20.49 ± 1.00	27	1.8731	0.1824
NO3	14.06 ± 2.87	10.81 ± 2.06	27	0.8644	0.3607
NH4	5.94 ± 1.34	4.07 ± 0.63	27	1.6688	0.2074
STN	0.34 ± 0.02	0.27 ± 0.02	27	6.2632	**0.0187**
STC	3.88 ± 0.21	3.09 ± 0.19	27	7.8593	**0.0092**

## Discussion

The use of cover crops to improve soil health is a key component of sustainable agriculture but the impact of different cover crop species on soil microbial communities remains poorly understood. This study evaluated the effect of 6 cover crop treatments on soil fungal communities relative to no cover crop treatment. Ascomycota, Mortierellomycota, and Basidiomycota were the most abundant fungal phyla identified in our soil samples which is consistent with previous studies [28, 47, 48]. These phyla inhabit a wide range of ecological niches and provide vital ecosystem services. Ascomycota play an important role in degradation of recalcitrant lignin-containing litter [49] and may outcompete other phyla for food and space as it has many genes related to stress tolerance and resource uptake [50]. Mortierellomycota enhance plant health by promoting phytohormones production (e.g., gibberellins, and indoleacetic acid), producing antagonist substances such as arachidonic acid that elicit phytoalexins in plants, and providing plants with nutrients such as phosphorus [24]. Basidiomycota, the most complex and evolutionarily advanced group of this kingdom contains important symbionts of other organisms and play a vital role in carbon cycling [51].

Consistent with our hypothesis, cover crop treatment affected both the richness and community structure of soil fungal communities, with the effect of cover crops on fungal richness being more widespread compared to the effect on community structure. All cover crop treatments enhanced soil fungal community richness and one cover crop treatment (CRYE) altered the soil fungal community structure relative to REF treatment. Previous studies on the effect of cover crops on soil fungal communities have yielded mixed results. A study evaluating the effect of 12 cover crops on soil fungal communities observed significant effect of cover crops on fungal community structure but not diversity [27]. Legume cover crops and soybean enhanced soil fungal diversity and altered the fungal community structure [28]. Plots with corn-soybean rotation with pennycress cover crop following corn had significantly different fungal communities relative to plots with corn-soybean rotations with no cover crop or plots with a corn-soybean rotation with an annual winter rye following both corn and soybean [52]. Alfalfa cover crop enhanced soil fungal richness, reduced fungal diversity, and altered the fungal community structure [53]. Monocultures of cereal rye and forage radish had unique core OTU composition while a mixture of three cover crop species (Australian winter pea, common medium red clover, and cereal rye) had distinct arbuscular mycorrhizal (AM) fungal community composition [27]. Oat, radish, rye, and rye-radish mixture affected soil fungal community structure but had no effect on fungal community richness [26]. Other studies have shown that cover crop mixtures may increase soil microbial diversity by creating diverse ecological niches that can be exploited by different microbes such as higher litter biomass and better C:N ratio [47, 54]. The varying effects of cover crops on fungal communities reported from these studies are expected since factors such as main crop identity, soil management practices, and soil types have been shown to influence soil microbial diversity and community structure [55, 56].

We found 48 fungal ASVs that were differentially abundant between CRYE and REF treatments. The most obvious difference between CRYE and REF treatments was the enrichment of *Fusarium* spp. and suppression *Mortierella* spp. in the REF relative to CRYE treatment. *Fusarium* is a diverse genus of fungi consisting of species from multiple guilds (pathotrophs, saprotophs, and symbiotrophs). The genus is known to contain some of the world’s most economically destructive plant pathogens [57]. CRYE had significantly higher soil fungal diversity and richness relative to REF treatment which can potentially contribute to the suppression of pathogenic fungi such as those within the genus *Fusarium* due to recruitment of *Mortierella* and other beneficial fungi [28, 58]. Since pathogen detection was not the objective of this study, we did not identify the *Fusarium* species detected in our soil samples. Additional studies are needed to determine how cover crop species influence the populations of beneficial and pathogenic members of this genus. Such studies may reveal the potential to harness CRYE cover crop to limit *Fusarium*-related economic damage if it can suppress pathogenic members of *Fusarium* sp. Interestingly, the relative abundance of *Fusarium* species in PCRO plots was identical to that of REF plots and much higher compared to other cover crop treatments. These findings suggest that PCRO cover crop mixture may increase the risk of *Fusarium*-related diseases in the main crop, but further studies are needed to determine whether the enriched members of genus *Fusarium* included the pathogenic species. Moreover, PCRO cover crop also increased the relative abundance of Order Mortierellales including genus *Mortierella* which is known to promote plant health [24]. Thus, although PCRO was association with high prevalence of *Fusarium* (which may include pathogenic species), their cultivation may be beneficial in cropping systems that are less susceptible to *Fusarium*.

Cover crops may alter soil fungal diversity and composition in various ways. Cover crops can affect soil fungal communities by altering soil characteristics that are known to influence fungal communities such as soil moisture, pH, temperature, total nitrogen, total carbon, organic matter content, and soil minerals [28, 53, 59]. We found limited influence of cover crops on soil properties in this study as none of the 18 soil variables tested were significantly different between cover crop treatments. In addition, none of the 18 soil variables were significantly associated with soil fungal communities. These results suggest that the observed differences in soil fungal communities between cover crop treatments were likely influenced by other variables that were not measured in this study (e.g. temperature and soil moisture). Some cover crop species also produce compounds that may have variable effects on different fungal taxa. For example, brassicaceous cover crops such as WPEN produce high levels of glucosinolates that can be hydrolyzed to form isothiocyanates with antifungal properties [60, 61]. These chemicals may enhance fungal species richness by reducing the abundance of dominant sensitive taxa, providing an opportunity for tolerant rare taxa to flourish. However, the influence of glucosinolates on soil fungal richness was not evaluated in this study and should be considered in future studies. In addition, WPEN had no significant effect on fungal community structure, likely because isothiocyanates have a short half-life and can disappear from the soil a few days after release [62].

Soil depth altered soil fungal community structure but had no significant effect on fungal species diversity and richness. Of the 15 fungal ASVs that were differentially abundant between the two soil depths tested, 9 and 6 ASVs, respectively were enriched in 0–2 cm and 2–4 cm soil depth. The majority of ASVs that were enriched at 0–2 cm soil depth were cellulolytic ascomycetous fungi including *Fusarium*, a genus of fungal plant pathogens known for causing significant yield-losses and production of mycotoxins in cereal crops that are lethal to humans [63]. Cellulolytic fungi colonize crop residuals rapidly and are most abundant during early stages of litter decomposition [64]. Previous studies on the effect of soil depth on fungal diversity and community structure have yielded mixed results. Some studies failed to detect any significant effect of soil depth on fungal diversity, richness, and community structure [65, 66]. Other studies reported significant differences in fungal community structure across soil depths and a negative relationship between soil depth and fungal diversity and richness [33, 34]. Soil properties are known to play an important role in shaping fungal community structure across soil depths [67–69]. Thus, we attribute the differences in fungal community structure between the two soil depths to the observed variations in soil properties. We identified 7 soil physicochemical variables (Fe, Cu, Zn, Mn, ICP_P, ICP_K, and STN) that were significantly associated with fungal communities. Two of these factors (ICP-P and ICP-K) as well as STN, and STC were significantly higher at 0–2 cm depth compared to 2–4 cm suggesting they could be a limiting factor for microbial growth at lower soil depths.

In summary, we found significant response of soil fungal communities to cover crop treatments and soil depth. All cover crop treatments including CRYE, PCRO, and WPEN enhanced soil fungal community richness and one cover crop (CRYE) treatment altered the community structure. The fungal community structure was also distinct at the two soil depths that were examined. These findings improve our understanding of how different cover crop species influence the diversity and structure of fungal communities in agricultural soils. This knowledge will facilitate the selection of cover crop species that are well suited for a particular cropping system.
